# Phospholipase A2 Mediates Apolipoprotein-Independent Uptake of Chylomicron Remnant-Like Particles by Human Macrophages

**DOI:** 10.1155/2012/501954

**Published:** 2011-08-21

**Authors:** Mariarosaria Napolitano, Howard S. Kruth, Elena Bravo

**Affiliations:** ^1^Department of Cell Biology and Neurosciences, Istituto Superiore di Sanità, Viale Regina Elena 299, 00161 Rome, Italy; ^2^Section of Experimental Atherosclerosis, National Heart, Lung, and Blood Institute, National Institutes of Health, Bethesda, MD 20892-1422, USA

## Abstract

Apolipoprotein E-receptor-mediated pathways are the main routes by which macrophages take up chylomicron remnants, but uptake may also be mediated by receptor-independent routes. To investigate these mechanisms, triacylglycerol (TG) accumulation induced by apolipoprotein-free chylomicron remnant-like particles (CRLPw/o) in human monocyte-derived macrophages was evaluated. Macrophage TG content increased about 5-fold after incubation with CRLPw/o, and this effect was not reduced by the inhibition of phagocytosis, macropinocytosis, apolipoprotein E function, or proteoglycan bridging. The role of lipases, including lipoprotein lipase, cholesteryl ester hydrolase, and secretory (sPLA2) and cytosolic phospholipase A2, was studied using [^3^H]TG-labelled CRLPw/o. Total cell radioactivity after incubation with [^3^H]TG CRLPw/o was reduced by 15–30% by inhibitors of lipoprotein lipase and cholesteryl ester hydrolase and by about 45% by inhibitors of sPLA2 and cytosolic PLA_2_ . These results suggest that macrophage lipolytic enzymes mediate the internalization of postprandial TG-rich lipoproteins and that sPLA_2_ and cytosolic PLA2, play a more important role than extracellular lipoprotein lipase-mediated TG hydrolysis.

## 1. Introduction

Lipids from the diet are absorbed from the intestine in chylomicrons, large triacylglycerol (TG)-rich lipoproteins, which are secreted into lymph and pass into the blood via the thoracic duct. The chylomicrons then undergo rapid lipolysis by lipoprotein lipase (LPL) in extrahepatic capillary beds, a process which removes some of the TG and leaves smaller chylomicron remnants which deliver the remaining dietary lipids to the liver [1]. It was believed for many years that chylomicron remnant size prevented their entrance into the artery wall and their subsequent interaction with macrophages. However, it is now clear that chylomicron remnants can penetrate and be retained within the subendothelial space as efficiently as low density lipoprotein (LDL) [2, 3]. In addition, apolipoprotein-B48-containing lipoproteins have been isolated from atherosclerotic plaques [4]. 

Chylomicron remnants have been shown to be taken up by several types of macrophages and to cause extensive TG and cholesterol accumulation leading to foam cell formation [5–8], further supporting the atherogenic role of this lipoprotein. The pathways mediating macrophage uptake of chylomicron remnants are apolipoproteinE (apoE)-dependent receptor-mediated processes involving the LDL receptor and the LDL receptor-related protein (LRP) [9–11]. However, several studies have found evidence that chylomicron remnant uptake pathways in these cells may be independent of the LDL receptor [6, 12] and apoE production [6]. Furthermore, Fujioka et al. [6] have reported that apolipoprotein-free remnant particles are taken up and promote lipid deposition in macrophages from apoE-deficient mice [6]. Thus, it seems likely that there are non-apoE-mediated receptor pathways which mediate the uptake of apolipoprotein-free chylomicron remnants by human macrophages. The aim of this study was to investigate these pathways and to evaluate whether secretory lipases are involved in their function.

## 2. Materials and Methods

### 2.1. Materials

Glycerol-tri[9,10(n)-^3^H]oleate (28 Ci/mmol), [1(3)-^3^H]glycerol (60 mCi/mmol), and [4-^14^C]cholesteryl-oleate (60 mCi/mmol) were obtained from NEN Life Science Products Inc., Boston, Mass, USA. Iscove's Modified Dulbecco's Medium (IMDM), fetal bovine serum (FBS), Ficoll-Paque, penicillin, and streptomycin were obtained from Hyclone Europe Ltd. CD14 MicroBeads and LS Separation Columns were purchased from Miltenyi Biotech. Goat antibody to human apoE and goat immunoglobulin G (IgG) were obtained from Biodesign (Bologna, Italy). Cytochalasin D, orlistat, brefeldin, manoalide, fatty acid-free bovine serum albumin (BSA), phorbol 12-myristate 13-acetate (PMA), heparinase I, heparinase III, MJ33, sodium chlorate, and various classes of lipids and solvents were purchased from Sigma Chemical Company (St. Louis, Mo, USA). methyl arachidonyl fluorophosphonate (MAFP) and haloenol lactone suicide substrate (HELSS) were purchased from Biomol International (Vinci-Biochem, Vinci, Italy). For lipid analysis of lipoprotein particles, enzymatic kits for the determinations of total (TCH) and free cholesterol (FCH) were obtained from WAKO (Test Medical, Zola, Italy) and that for TG from BPC (Rome, Italy).

### 2.2. Macrophages

Monocytes were isolated from human buffy coats as previously described [13]. Buffy coats from the blood of healthy donors were diluted 1 : 3 with phosphate-buffered saline (PBS) and layered on Ficoll-Paque. After centrifugation, white blood cells were collected and washed with PBS. CD14 MicroBeads were used for the positive selection of human monocytes from white blood cells. According to the manufacturer's instructions, 300–400 ×10^6^ total cells, magnetically labelled with CD14 MicroBeads, were applied to LS Separation columns, and the total effluent was discarded. Monocytes (CD14-positive fraction), flushed out of the column, were washed, and 1.5 × 10^6^ cells transferred to 22-mm dishes at a concentration of 8 × 10^5^ cells/mL and cultured in IMDM containing 15% FBS. The purity of isolated monocytes, monitored by specific flow cytometric analysis for CD14, ranged 95–97%. The differentiation process from monocytes to macrophages was monitored by the increased expression of CD71 antigen. The experiments were performed with human monocyte-derived macrophages (HMDM) 10 days after plating. For experiments involving the measurement of lipid accumulation, CRLP were incubated with HMDM for 24 h, so that there was sufficient uptake to allow accurate determination of TG and cholesterol in the cells. Shorter incubation times (5-6 h) were used for experiments involving radioactivity, as these techniques are more sensitive.

Cultures of the J774.2 murine macrophage-like cell line (J774) were obtained from the American Type Culture Collection (Rockville, Md, USA). Cells were maintained in DMEM supplemented with penicillin (100 U/mL), streptomycin (100 *μ*g/mL), glutamine (2 mM), and 10% FBS at 37°C in a humidified atmosphere of 95% air/5% CO_2_. For experiments, cells (at passage 6–10) were seeded into 22-mm dishes at a concentration of 10^5^ cells/mL and used on the 3rd day of culture.

### 2.3. Preparation of CRLP

Chylomicron remnant-like particles (CRLP) containing TG as the major lipid class were prepared by sonication of a lipid mixture followed by ultracentrifugation [14]. A lipid mixture containing 70% triolein (18 : 1), 2% cholesterol, 3% cholesteryl ester, and 25% phospholipids (70.5% phosphatidylcholine, 11% phosphatidylethanolamine, 6.9% lysophosphatidylcholine, 6.5% sphingomyelin, 2.6% phosphatidylinositol, and 2.6% phosphatidylserine) was sonicated in 0.9% NaCl in Tricine buffer (20 mM, pH 7.4) for 20 min at 37°C with a power giving an amplitude range, quoted as total peak to peak moments of 22–24 *μ*m (Branson 250/450 sonifier). For the preparation of CRLPw/o with labelled TG, before sonication, 100 *μ*Ci of glycerol-tri[^3^H] oleate ([^3^H]TG) was added to the lipid mixture. After sonification, the density of the emulsion was increased to 1.21 g/mL with KBr, layered under a step-wise density gradient and centrifuged at 17,000 g for 20 min at 20°C. The upper layer was discarded and replaced with an equal volume of KBr (d 1.006 g/mL), and the centrifugation was repeated at 70,000 g for 1 h at 20°C. The particles were harvested from the top layer, dialyzed against medium without FBS, but containing penicillin/streptomycin. These CRLP (CRLPw/o), which do not contain apolipoproteins, were used for experiments within 2 days of their preparation. For the preparation of lipid particles containing apoE (CRLP+), CRLPw/o were incubated with human plasma as previously described [13]. Previous analysis by SDS-PAGE has shown that CRLP prepared in this way contain apoE and no other apolipoproteins [11]. The band corresponding to apoE was not detected in lipid particles prior to incubation with plasma, nor in the top fraction from plasma centrifuged in the absence of lipid particles, indicating that the CRLP acquired apoE during the incubation.

### 2.4. Negative Staining Electron Microscopy of CRLP

Samples were diluted with distilled water to achieve a satisfactory concentration of lipid particles for negative staining. A 200-mesh nickel grid with a type-B carbon support film (Ted Pella, Redding, Calif, USA) was incubated with a drop of diluted sample. After 10 minutes, the grid was drained with filter paper and stained with 2% phosphotungstic acid (pH = 4) for 2 minutes. The grid was drained and photographed with a Jeol 1200 transmission electron microscope.

### 2.5. Cellular Assay of Cholesterol and TG

After incubations, cells were washed 3 times with PBS and harvested from wells by scraping into 500 *μ*L of distilled water. An aliquot of cellular suspension was utilized to determine protein content by Lowry's method [15], using BSA as a standard. After the extraction of cellular lipids [16], the TCH and TG content of cells was determined by fluorimetric methods according to Gamble et al. [17] and Mendez et al. [18], respectively. Each determination was performed in duplicate.

### 2.6. Assay of TG Synthesis

Synthesis of TG and PL was evaluated by determining the incorporation of [^3^H]glycerol into TG and PL as previously described [13]. HMDM were incubated for 5 h at 37°C in serum-free IMDM containing 80 *μ*g cholesterol/mL CRLPw/o in the presence of [^3^H]glycerol (4 *μ*Ci/mL, 20 *μ*M). After the incubations, HMDM were washed 3 times with PBS, and cell lipids were extracted with hexane/isopropanol; 3 : 2, v/v. [^14^C]CE was added as an internal standard and the lipids classes were separated by thin layer chromatography on silica gel (Merck, Germany) developed in hexane/ether/acetic acid (70 : 30 : 1, v/v/v). Radioactivity associated with the bands corresponding to [^3^H]TG and [^3^H]PL were scraped from the plates and assayed for radioactivity in an LS5000 Beckman liquid scintillation counter.

### 2.7. Statistical Methods

Repeated Measure Analysis of variance (ANOVA) and multiple comparisons using the Tukey-Kramer Multiple Comparison Test or Student's paired *t*-test were used to evaluate significant differences in the means between groups. *P* < 0.05 was considered significant.

## 3. Results

### 3.1. Characterization of CRLP

**Figure 1:**
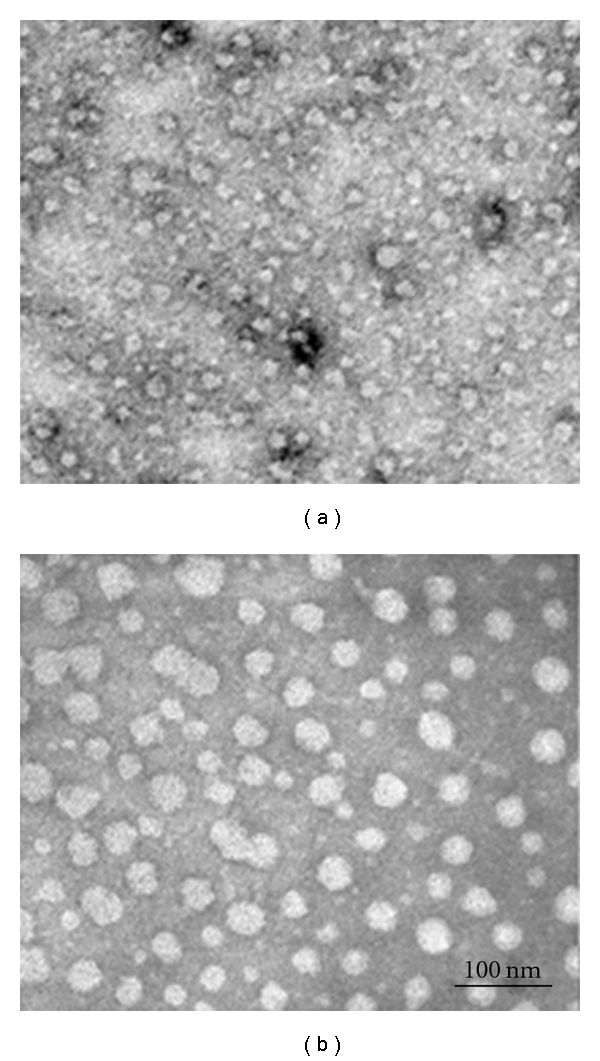
Negative staining electron microscopy (EM). Negatively stained CRLPw/o (b) and CRLP+ (a). The particle diameter expressed as mean ± SD was 34 ± 5 nm for CRLPw/o (190 particles) and 24 ± 7 nm for CRLP+ (160 particles).

Biochemical characterization of CRLPw/o and CRLP+ (Table 1) showed that CRLPw/o and CRLP+ differ in their TG/TCH (9.68 ± 2.88 versus 4.95 ± 2.30, respectively; *P* < 0.005) and FCH/TCH (0.52 ± 0.30 and 0.21 ± 0.11, resp.; *P* < 0.05) molar ratios. As the TG concentration was not different between CRLPw/o (8.41 ± 2.9 mM) and CRLP+ (7.42 ± 3.79 mM), much of the difference between the lipid ratios are attributable to a decreased TCH content in CRLP-w/o in comparison with CRLP+ (0.87 ± 0.57 mM versus 1.79 ± 0.88 mM, *P* < 0.005). These changes in biochemical composition were accompanied by differences in the size of the particles. The average particle diameter determined from electron microscopy of CRLPw/o (Figure 1(b)), and CRLP+ (Figure 1(a)) was 34 ± 5 nm and 24 ± 7 nm, respectively. Preparations of [^3^H]-TG CRLPw/o produced particles with a specific activity of 2780 and 3810 dpm/nmol of fatty acid in the 2 different preparations used for the 3 experiments. Radioactivity distribution in the lipids carried by the particles was similar in the 2 preparations. More than 90% of the radioactivity carried by [^3^H]-TG CRLP was associated with TG, while the radioactivity associated with PL, free fatty acids, cholesteryl ester, and the sum of radioactivity recovered from the TLC plate but not associated with these bands was 2.8, 2.1, 0.4, and 4.7 %, respectively.

### 3.2. Role of Plasma-Derived Factors in Macrophage Lipid Accumulation Induced by CRLP

**Figure 2:**
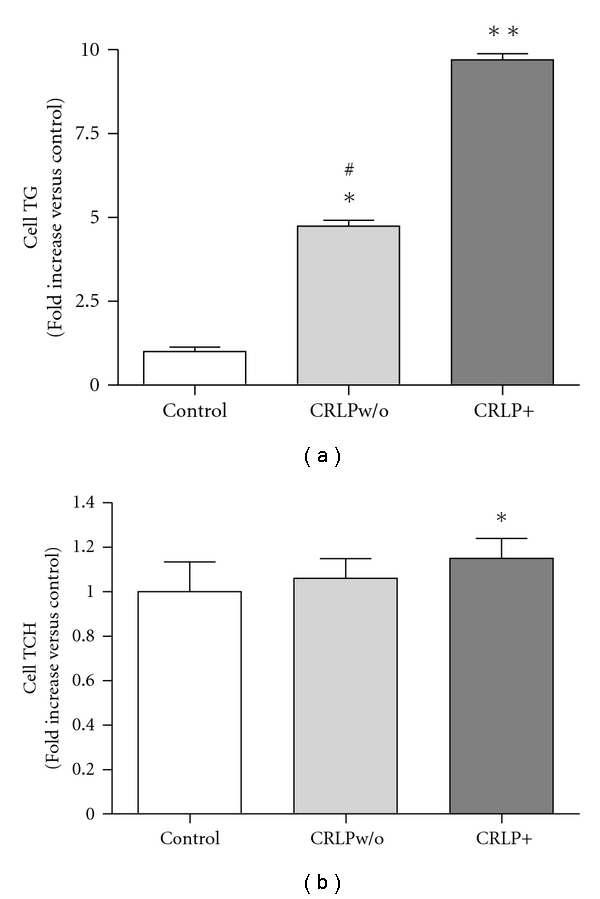
Effect of plasma treatment of CRLP on macrophage lipid accumulation induced by CRLP. HMDM were incubated for 24 h in serum-free medium without the addition of CRLP (control) or with 80 *μ*g cholesterol/mL of either CRLP+ (contains apoE) or CRLPw/o (lacks apoE). (a) Cellular TG; (b) total cholesterol (TCH) content was determined by fluorimetric assay (mean ± SD, *n* = 6). Results are expressed as increase of lipid content with respect to the control. *P < 0.05, **P < 0.001 versus control; ^#^ *P *< 0.05 versus CRLP+.

To investigate the role of plasma-derived factors in the induction of human macrophage lipid accumulation by chylomicron remnants, we measured macrophage TG and TCH of macrophages incubated with either CRLP+ or CRLPw/o in comparison to macrophages incubated without the lipid particles. The small consistent increase (12%) of cell cholesterol (Figure 2(a)) induced by CRLP+ (89.4 ± 4.5 nmol/mg protein, *n* = 6; *P* < 0.05 versus control w/o) in comparison with the control (79.1 ± 4.9 nmol/mg protein), was not observed with CRLPw/o (83.9 ± 7.4 nmol/mg protein). The presence of CRLP+ induced almost a 10-fold increase in TG macrophage content (47.8 ± 15.8 and 464.3 ± 190.2 nmol/mg protein in the absence or presence of CRLP+, resp.), and this was reduced to about 5 fold (226.3 ± 93.1 nmol/mg protein), (Figure 2(b)), when CRLPw/o particles lacking apolipoproteins were used. Thus, the increase in macrophage TG content after incubation with CRLPw/o remained substantially higher than the control (*P* < 0.05), indicating that CRLPw/o can induce macrophage lipid accumulation independent of plasma-derived apolipoproteins.

### 3.3. Role of Phagocytosis and Macropinocytosis in Macrophage Internalization of CRLPw/o

**Figure 3:**
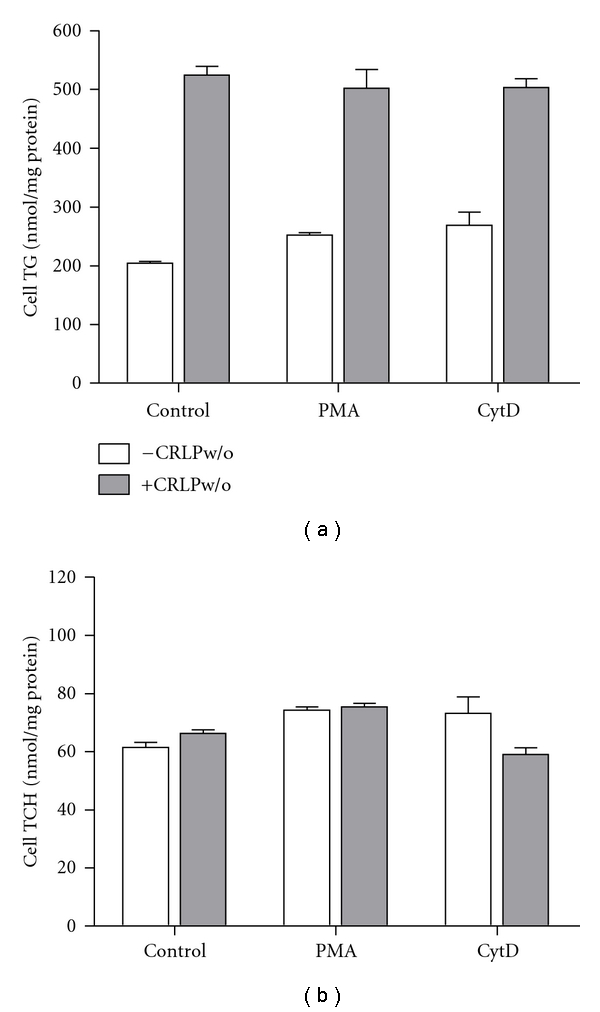
Effect of cytochalasin D and PMA on macrophage lipid accumulation. HMDM were incubated 24 h with 80 *μ*g cholesterol/mL CRLPw/o or without CRLPw/o in the presence of 2 *μ*g/mL cytochalasin D (CytD), or 1 *μ*g/mL PMA both dissolved in DMSO or DMSO alone (control). Macrophage triacylglycerol (TG) and total cholesterol (TCH) content (nmol lipid/mg protein) were determined by fluorimetric assay (mean ± SD, *n* = 4).

Macrophages are professional phagocytes which carry out two related uptake processes, phagocytosis, and macropinocytosis [19], both of which may be involved in atherosclerosis development [20, 21]. Phagocytosis and macropinocytosis can be blocked by cytochalasin D, and macropinocytosis can be induced in some macrophage phenotypes by treatment with phorbol esters such as PMA [20]. Thus, we investigated the function of these pathways on CRLPw/o uptake by incubating HMDM with CRLPw/o 20 *μ*g (results not shown) or 80 *μ*g cholesterol/mL) in the presence of cytochalasin D (2 *μ*g/mL), or PMA (1 *μ*g/mL), or the vehicle alone (control). The results (Figure 3) show that the changes in cellular TG and TCH content caused by CRLPw/o were not modified by cytochalasin D or PMA, suggesting that, over the concentration range of CRLP tested, phagocytosis and macropinocytosis are not directly involved in CRLPw/o-induced macrophage lipid accumulation.

### 3.4. Effect of Inhibition of Macrophage Secretion on TG Accumulation

Macrophages display a wide range of functions and secrete many factors potentially affecting lipoprotein metabolism (i.e., apoE and lipases) that could mediate uptake of CRLPw/o. To evaluate if factors secreted by macrophages contribute to the internalization of CRLPw/o, we added CRLPw/o (80 *μ*g cholesterol/mL) to macrophages that had been preincubated for 3 h with 0 (control), 5 and 15 *μ*g/mL brefeldin, an early stage inhibitor of the secretory pathway [22]. Incubation was then continued for 24 h in the presence of brefeldin. Macrophage TG content (542 ± 142 nmol TG/mg protein in the absence of brefeldin) was significantly (*P* < 0.05, *n* = 6) reduced by about 39% and 31%, respectively, by 5 and 15 *μ*g/mL of brefeldin (434 ± 139 and 489 ± 126 nmol TG/mg protein with 5 and 15 *μ*g/mL of brefeldin, resp.) compared with the control, suggesting that secretory factors produced by macrophages contribute to TG accumulation induced by CRLPw/o in macrophages.

### 3.5. Macrophage apoE Secretion and Internalization of CRLPw/o

The absence of plasma-derived apoE in CRLPw/o reduced but did not prevent macrophage lipid accumulation induced by the lipid particles (Figure 2). However, apoE is secreted in large amounts by human macrophages [23]; thus, CRLPw/o uptake could be mediated via the acquisition of the apolipoprotein during the incubation. To test this hypothesis, we measured the effects of an apoE antibody (apoE-Ab) on macrophage TG accumulation induced by CRLPw/o. For this purpose, HMDM were incubated for 24 h with CRLPw/o (80 *μ*g cholesterol/mL) in the presence of 100 *μ*g/mL of apoE-Ab or control IgG isotype. The TG content of macrophages at the end of incubations with apoE-Ab (708.9 ± 152.6 nmol/mg protein) was not different from incubations with IgG (842 ± 310 nmol/mg protein) or in the absence of antibody (control: 703.5 ± 213.5 nmol/mg protein) (*n* = 3), indicating that apoE secreted by macrophages does not account for the uptake of the apoE-free CRLPw/o.

To further ascertain whether the secretion of apoE has a role in CRLPw/o lipid uptake, we carried out experiments with J774 cells, a murine macrophage cell line which secretes extremely low levels of apoE [24]. In control J774 macrophages, similar to the results with HMDM, CRLPw/o (80 *μ*g cholesterol/mL) induced a significant increase in TG content (without CRLPw/o, 132.9 ± 21.9; with CRLPw/o 230.8 ± 10.9 nmol/mg protein; *P* < 0.05, *n* = 3) with no significant change in cholesterol content. Thus, the relative lack of apoE secretion did not prevent J774 macrophages from accumulating TG in the presence of CRLPw/o, confirming that apoE is not necessary for the uptake of these particles by the cells.

### 3.6. Role of Proteoglycan Bridging in Lipid Accumulation Induced by CRLPw/o

**Figure 4:**
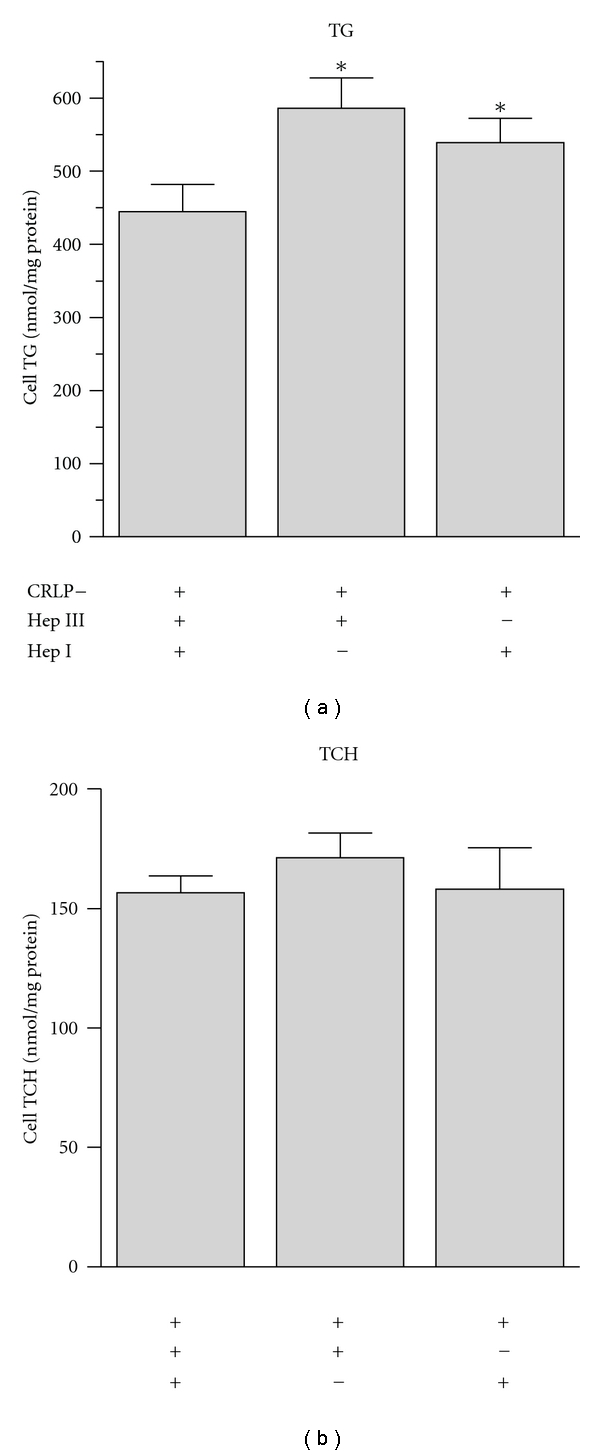
Role of proteoglycan-mediated bridging in macrophage lipid accumulation. HMDM were incubated 1 h in serum-free medium before treatment with 33 U/mL heparinase III (Hep III) or 33 U/mL heparinase I (Hep I) in combination with 50 mM Na chlorate. Both untreated and treated macrophages were then incubated 24 h with 80 *μ*g cholesterol/mL CRLPw/o. Treatments were continued during the incubations. Macrophage triacylglycerol (TG) and total cholesterol (TCH) contents are reported as nmol/mg protein (means ± SD, *n* = 4). *P < 0.005 versus control.

The interaction of lipoproteins with arterial proteoglycans facilitates the retention and the metabolism of TG-rich lipoproteins. In particular, proteins secreted by macrophages, such as apoE, lipoprotein lipase (LPL), and the secretory phospholipase A2 (sPLA_2_), independently of their function, can act as structural cofactors facilitating cellular uptake of whole lipoprotein particles. These molecules can bridge between lipoproteins and heparan sulfate proteoglycans, concentrating lipoproteins in the vicinity of receptors or promoting entry of lipoproteins during the process of cell surface proteoglycan internalization [25, 26]. If this process was involved in CRLPw/o internalization by macrophages, therefore, the inhibition of the interaction with proteoglycans would be expected to impair their uptake. To test this hypothesis, HMDM, untreated (control) or pretreated for 1 h with heparinase I (33 U/mL) [27] or heparinase III (33 U/mL), both with 50 mM Na chlorate to inhibit proteoglycan synthesis [28], were further incubated for 24 h with CRLPw/o (80 *μ*g cholesterol/mL). As reported in Figure 4, a rise (rather than a decrease) in TG content was induced by both heparinase III and heparinase I (586 ± 71 and 539 ± 58 nmol/mg protein, resp.) in comparison with the control group (445 ± 64 nmol/mg protein). Thus, the disruption of cell surface proteoglycans with heparinase did not decrease CRLP TG uptake by macrophages, indicating that the function of LPL, and other secreted proteins in bridging with proteoglycan is not involved in CRLPw/o internalization by macrophages.

### 3.7. Role of Macrophage Lipase Activities on the Internalization of TG Carried by CRLPw/o

**Figure 5:**
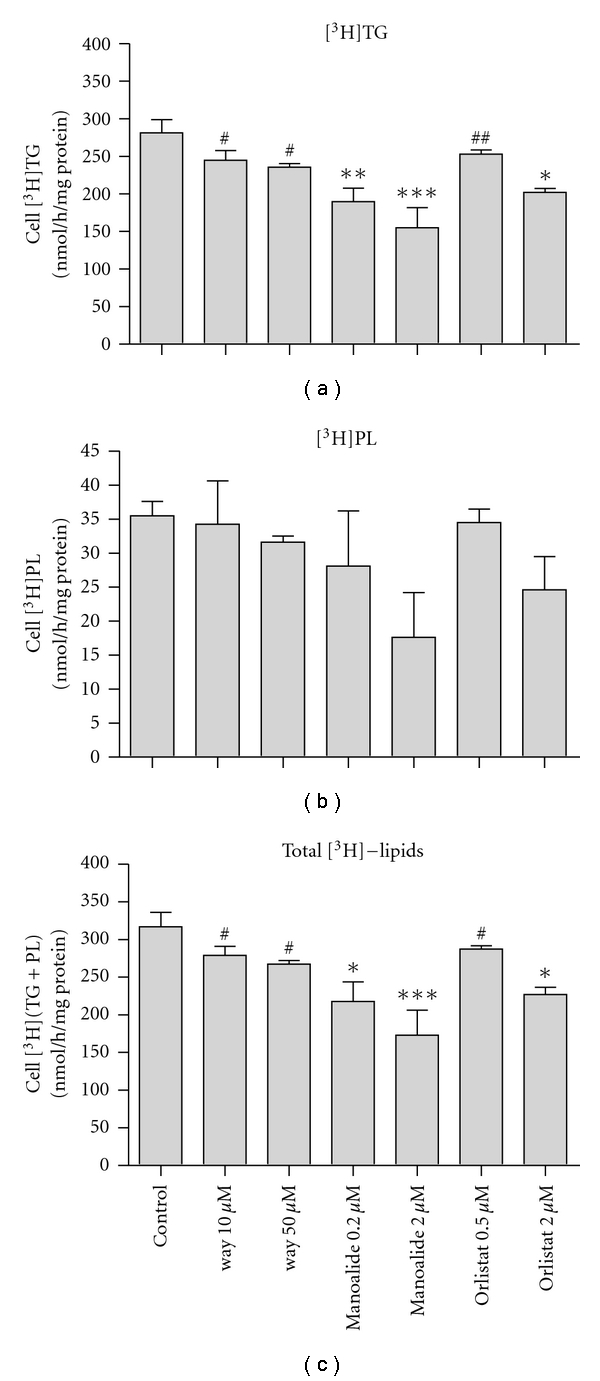
Role of macrophage-secreted lipases on [^3^H]TG-CRLPw/o internalization. Cell radioactivity associated with macrophage lipids was determined after a 6-h incubation of HMDM with 80 *μ*g cholesterol/mL of [^3^H]TG-CRLPw/o in the presence of 10 and 30 *μ*M way121.989 (way), 0.2 and 2 *μ*M of manoalide, 0.2 and 2 *μ*M of orlistat or in the absence of any inhibitor (control). Radioactivity associated with free fatty acids and cholesteryl ester was negligible, and that associated with macrophage triacylglycerol ([^3^H]TG, (a)), phospholipid ([^3^H]PL, (b)), and [^3^H](TG + PL) (c) is reported as nmol/h/mg protein (*n* = 3). *P < 0.05, **P < 0.01, ***P < 0.001 versus control; ^#^ *P* < 0.05, ^##^ *P* < 0.01 versus manoalide 2 *μ*M.

The experiments above show that CRLPw/o induces the accumulation of TG but do not provide information about the mechanism involved. However, the data obtained with brefeldin suggests that macrophage-secreted factors may function in CRLPw/o TG uptake. Among the proteins secreted by macrophages, there are a number of lipases, including LPL, sPLA_2_, and cholesteryl ester hydrolase [29–32]. Secreted LPL has been shown to have an important function in the uptake of fatty acids derived from the extracellular lipolysis of TG carried by TG-rich lipoproteins [29]. In view of this, we examined whether the lipases produced by macrophages contribute to TG uptake from CRLPw/o. Macrophage sPLA_2_ cleave phospholipid (PL) fatty acids in the sn-2 position [31], and, while fatty acids released by cholesteryl ester hydrolase secreted by macrophages derive mainly from cholesteryl ester, TG and PL may also function as substrates [31]. The fatty acids liberated by the catalytic action of extracellular lipase, however, could supply substrate for cellular TG synthesis, even without internalization of whole CRLPw/o. Alternatively, fatty acids released by these lipases could occur after the internalization of CRLPw/o. In order to evaluate whether macrophage lipolytic activities contribute to the internalization of intact TG carried by CRLPw/o, we used an experimental approach involving radiolabelled CRLPw/o particles. HMDM were incubated with [^3^H]TG-CRLPw/o (80 *μ*g cholesterol/mL) in the presence of different lipase inhibitors, and cell incorporation of CRLPw/o lipid was evaluated by measuring the radioactivity recovered in macrophage lipids. To inhibit cholesteryl ester hydrolase, LPL and sPLA_2_, way121.989 [33], orlistat [34], and manoalide [35], respectively, were used. After incorporation into macrophages, radioactivity transported by [^3^H]TG is redistributed into cellular fatty acids. To assess this, at the end of incubations with [^3^H]TG-CRLPw/o, lipids were extracted from the cells and the radioactivity associated with TG, PL, free fatty acids, and cholesteryl esters was determined [7]. Most of the radioactivity was found in macrophage [^3^H]TG and, much less, about 1/10, was in the [^3^H]PL fraction. The radioactivity associated with free fatty acids and cholesteryl esters was negligible and was not taken into account in further analysis of the data. Thus, the total radioactivity taken up by macrophages was calculated as the sum of [^3^H]TG + [^3^H]PL. The results are shown in Figure 5. None of the inhibitors had any significant effect on the incorporation of radioactivity into PL (central panel), although all of them tended to decrease the total radioactivity taken up by macrophages, compared with macrophages incubated without inhibitors (control). However, the reductions were significant in comparison to the control only for manoalide (*P* < 0.05 and *P* < 0.001 at 0.2 and 2 *μ*M of manoalide, respectively; *n* = 3) and for the higher concentration (2 *μ*M) of orlistat (*P* < 0.05; *n* = 3). The decreased total radioactivity incorporated by macrophages treated with both concentrations of manoalide reflected the lower macrophage accumulation of [^3^H]TG (upper panel) in these conditions, in comparison with either the control or way121.989. Thus, the internalization of TG carried by CRLPw/o is mainly dependent on sPLA_2_ activity, but not cholesteryl ester hydrolase or LPL.

### 3.8. Role of Lipase Activities in TG and PL Synthesis after Uptake of CRLP by Macrophage

**Figure 6:**
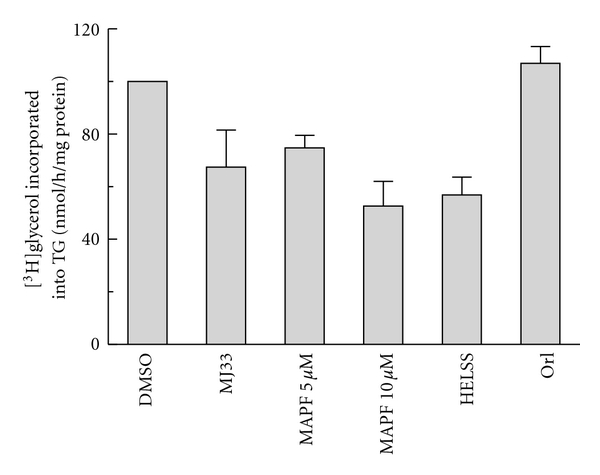
Role of macrophage lipase activities in TG synthesis. HMDM were preincubated for 2 h with or without 10 *μ*M MJ33, 5 *μ*M and 10 *μ*M MAFP [36], 5 *μ*M HELSS, and 2 *μ*M orlistat. Incubation was then continued for 5 h in presence of CRLPw/o (80 *μ*g cholesterol/mL) in the presence of [^3^H]glycerol (4 *μ*Ci/mL, 20 *μ*M)to evaluate the incorporation of [^3^H]glycerol into TG. Radioactivity associated with triacylglycerol ([^3^H]TG) is reported as nmol/h/mg protein (*n* = 3). *P < 0.05 versus control.

To assess how lipases affect lipid synthesis in HMDM after uptake of CRLPw/o, cells were preincubated for 2 h with or without inhibitors of sPLA_2_ (MJ33 10 *μ*M), cytosolic PLA_2_ (MAFP, 5 *μ*M and 10 *μ*M) [36], calcium-dependent cytosolic PLA_2_ (HELSS, 5 *μ*M) [37] and LPL (orlistat, 2 *μ*M). Incubation was then continued for 5 h in the presence of CRLPw/o (80 *μ*g cholesterol/mL) to evaluate the incorporation of [^3^H]glycerol into TG and PL (Figure 6). 

PL synthesis was not significantly affected by any of the inhibitors tested (data not shown), but HELSS and MAFP at a concentration of 10 *μ*M were found to inhibit TG synthesis (*P* < 0.05), while orlistat, MJ33, and 5 *μ*M MAFP had no significant effect. These results suggest that cytosolic and calcium-dependent PLA_2_, but not sPLA_2_ or LPL, play a part in the regulation of TG synthesis after uptake of CRLPw/o by human macrophages.

## 4. Discussion

Chylomicron remnants can induce TG accumulation and foam cell formation [7–10]; however, the interactions between these postprandial lipoproteins and macrophages are poorly understood. Much evidence suggests that both receptor-dependent and -independent mechanisms function in lipid accumulation, as apolipoproteins may not be necessary for receptor interaction [6, 38] or induction of macrophage lipid accumulation [6, 12, 39]. However, studies and manipulation with chylomicron remnants are methodologically limited. There are marked difficulties in the preparation of postprandial lipoprotein fractions not contaminated by other lipoproteins. Thus, although studies performed with lipoprotein models devoid of apolipoproteins may not reproduce physiological conditions, these models help to clarify the apolipoprotein receptor-independent mechanisms, which contribute in vivo to the accumulation of lipids in the vessel wall. We focused the current investigation on the mechanisms of internalization involved in lipid accumulation caused by apolipoprotein-free CRLPw/o. Macrophages are now known to have both pro- and anti-inflammatory properties; inflammation is essential for protection against pathogens, but healing requires the deleterious effects on the tissues to be suppressed. This dual role is facilitated by alternative activation of the cells into a pro- (M1) or anti-inflammatory (M2) phenotype [40]. Since our macrophage model is obtained in vitro in the absence of any stimulus, taking into account the limitations of a cell model, we believe it resembles more closely the classically polarized, round-shaped activated M1 macrophages.

Macrophages are now known to have both pro- and anti-inflammatory properties; inflammation is essential for protection against pathogens, but healing requires the deleterious effects on the tissues to be suppressed. This dual role is facilitated by alternative activation of the cells into a pro- (M1) or anti-inflammatory (M2) phenotype [40]. Since our macrophage model is obtained in vitro in the absence of any stimulus, taking into account the limitations of a cell model, we believe it resembles more closely the classically polarized, round-shaped activated M1 macrophages.

We found that the incubation of CRLPw/o with plasma induces some changes in compositions of these lipid particles including the acquisition of apolipoproteins [11], as well as the decrease of change of the ratios TG/TCH and FCH/TCH (Table 1). Despite their similar TG concentrations, CRLPw/o contain less TCH with a larger proportion of FCH (about 50%), which probably functions to stabilize the surface of these particles devoid of apolipoproteins. Thus, in comparison with CRLP+, CRLPw/o are larger buoyant particles (Figure 1), stabilized at their surface by a higher number of FCH molecules.

The lack of apolipoproteins on CRLPw/o did not prevent a significant increase of TG in both HMDM and J774 cell line macrophages during incubation with CRLPw/o. CRLPw/o induced TG accumulation independently of both phagocytosis and macropinocytosis. Macropinocytosis and phagocytosis are related but independent actin-dependent processes functioning in macrophages [19] and can contribute to foam cell formation by facilitating lipid accumulation. In particular, macropinocytosis, which functions in uptake of particles in the fluid phase, has been shown to mediate the induction of foam cell formation by LDL [20]. However, macropinocytosis is particularly efficient at high levels of lipoprotein that could not be achieved with CRLP in the current investigation due to experimental limitations. Chylomicron remnants are specialized to transport high quantities of TG, which at elevated concentrations induce cell detachment and toxicity [6, 41, 42]. We also found that CRLPw/o induce slight cell detachment at a concentration higher than 80 *μ*g cholesterol/mL (data not shown). Thus, in our experimental conditions, conclusions about the lack of a role of macropinocytosis and phagocytosis involvement in CRLP processing are limited to the concentrations that we were able to test.

A possible mechanism for macrophage uptake of CRLPw/o is that they acquire macrophage-secreted apoE, which could lead to whole lipid particle uptake mediated by macrophage apoE-dependent receptors [43]. However, an anti-apoE antibody had no effect on TG accumulation induced by CRLPw/o in HMDM, suggesting that apoE is not necessary for CRLPw/o-mediated lipid accumulation in these cells and this conclusion was supported by the finding that CRLPw/o induced TG accumulation in J774 macrophages, a murine cell line which does not secrete apoE [24]. These results are in agreement with the observations of Fujioka et al. [6], who found that macrophages from apoE-deficient mice internalize apolipoprotein-free remnant particles. 

LPL and PLA_2_, as well as apoE secreted by macrophages, contribute to lipoprotein processing by acting as bridges between lipoproteins and heparan sulfate proteoglycans [25, 26]. However, our experiments exclude the possibility that extracellular lipases or other proteins that may act in this way are involved in macrophage CRLPw/o processing. Treatment with either heparinase I or heparinase III, which cause a loss of surface proteoglycans, increased CRLPw/o-induced macrophage TG accumulation (Figure 4), suggesting that surface proteoglycans may hinder rather than promote macrophage uptake of CRLPw/o.

The observation that the inhibition of macrophage secretion by brefeldin decreases CRLPw/o induced TG accumulation (Figure 3) focused our attention on macrophage secretory products. Although impairment of proteoglycans, apoE or LPL functions did not prevent TG accumulation, the inhibition of sPLA_2_ markedly reduced the internalization of CRLPw/o, indicating a role for this enzyme in the metabolism of TG-rich lipoproteins. As the free fatty acids released after extracellular lipolysis enter cells and are re-esterified, contributing to an increase in cell TG content, we expected that the LPL inhibitor orlistat would cause a reduction in cellular TG accumulation induced by CRLPw/o. Surprisingly, in contrast to what has been reported for VLDL and chylomicron remnants [13, 29, 43], the experiments with radiolabelled particles showed that LPL is not a major factor involved in the uptake of [^3^H]TG in CRLP devoid of apolipoproteins. The higher concentration of orlistat decreased the internalization of the [^3^H]TG-CRLPw/o (−29%) only when the total lipid radioactivity was taken into account (Figure 5(c)), while the changes in macrophage [^3^H]TG were not significant. On the other hand, the inhibition of sPLA_2_ activity by manoalide reduced in the incorporation of radioactivity into cellular [^3^H]TG as well as the total lipid radioactivity in a dose-dependent manner. The inhibition of [^3^H]TG-CRLP accumulation by HMDM induced by 0.5 *μ*M and 2 *μ*M of this inhibitor was about 32 and 45%, respectively, showing that, in contrast to LPL action, the catalytic activity of sPLA_2_ has a prominent role in macrophage accumulation of TG during incubation with CRLPw/o. 

This observation adds further details to the complex picture of the role of sPLA_2_ in the development of cardiovascular disease. The current opinion is that circulating members of the PLA_2_ family are positively associated with the pathogenesis of atherosclerosis by different mechanisms, including the generation of atherogenic particles [44], as well as the activation of several proinflammatory pathways [45]. Several physiological actions of sPLA_2_ are unrelated to their enzymatic activity; rather, they can be attributed to the engagement of specific receptors on target cells [46]. However, in this study, the hypothesis that manoalide influences [^3^H]TG-CRLPw/o metabolism by interfering with the specific binding of sPLA_2_ to a surface receptor seems unlikely, because the manoalide binds irreversibly to several lysine residues of the enzyme, inhibiting specifically its activity [35]. Furthermore, the macrophage sPLA_2_ subtypes comprise a diverse family of enzymes that catalyze the hydrolysis of the sn-2 ester bond of PL and glycerophospholipids but do not act on TG [31, 47, 48]. Thus, the effects of manoalide on cell [^3^H]TG recovery, in contrast to LPL, cannot be explained in terms of massive extracellular TG hydrolysis followed by intracellular re-esterification. In addition, as the radioactivity was specifically carried by [^3^H]TG in CRLPw/o, extracellular hydrolysis of PL did not contribute to the macrophage accumulation of [^3^H]TG observed in our study. However, since the particles contain 70% TG and 25% PL (of which about 85% can be substrates of PLA_2_), and TG contains 3 fatty acids that can be hydrolyzed and re-esterified, while PL contain only 2 fatty acids (with only 1 hydrolyzable by PLA_2_), about 1/10 of the fatty that can be re-esterified are in the PL and 9/10 in the TG. Thus, the finding that, after uptake of the radiolabelled CRLPw/o, approximately 1/10 of the radioactivity is found in phospholipids suggests that the whole particles are taken up by the cells and that phospholipids are then hydrolyzed and re-esterified. Overall, our results indicate that sPLA_2_ activity could contribute to CRLPw/o lipid internalization by inducing lipoprotein modifications which, in turn, increase the catabolism of the whole particle and/or selectively facilitate uptake of the TG moiety. For instance, Tietge et al. [49] reported that overexpression of sPLA_2_ enzymatic activity alters the structure and composition of high-density lipoprotein (HDL) particles, enhancing selective uptake of cholesteryl ester with the metabolic consequences of increased catabolism of HDL. Similarly, some changes to CRLPw/o induced by the catalytic activity of sPLA_2_ could expose or hide domains in the particles, which then could facilitate macrophage uptake of the TG carried by CRLPw/o. More studies are needed, however, to substantiate this idea.

Although our data indicate a role for sPLA_2_ in the internalization of CRLPw/o, the inhibition of the enzyme did not affect the synthesis of TG from glycerol in HMDM after uptake of the particles (Figure 6). Interestingly, however, the results of this set of experiments indicate the involvement of cytoplasmatic PLA_2_ and in particular of Ca2+-dependent PLA_2_ in macrophage CRLPw/o metabolism. As MAFP is an irreversible inhibitor of of both calcium-dependent and calcium-independent cytosolic phospholipase A_2_, but not secretory phospholipase A_2_ [36], the finding that TG synthesis was decreased in the presence of this inhibitor supports the hypothesis that cytosolic PLA_2_ activity plays a role in CRLPw/o processing. In particular, our results directly implicate the Ca2+-dependent PLA_2_ activity, since HELSS, a direct and irreversible inhibitor of this enzyme [37], reduced the synthesis of TG induced by CRLPw/o in a similar manner to MAFP (Figure 6).

In conclusion, this study shows that sPLA_2_ plays a role in the extensive macrophage TG accumulation promoted by TG-rich chylomicron remnant-like lipoproteins. Furthermore, this macrophage TG accumulation occurs independently of apolipoprotein-mediated receptor interactions, supporting the concept that more attention should be paid to pathways for macrophage lipid internalization not mediated by apolipoprotein-receptor interactions.

## Figures and Tables

**Table 1:** Biochemical characterization of chylomicron remnant-like lipid particles (CRLP) incubated without (CRLPw/o) or with plasma (CRLP+). Total cholesterol (TCH), triacylglycerol (TG), and free cholesterol (FCH) are expressed as means ± SD (*n*).

	TCH	TG	FCH	TG/TCH	FCH/TCH
	(mM)	(mM)	(mM)	(molar ratio)	(molar ratio)
CRLPw/o	0.87 ± 0.57 (20)**	8.41 ± 2.90 (20)	0.45 ±0.21 (7)	9.68 ± 2.88 (20)**	0.52 ± 0.30 (7)*
CRLP+	1.79 ± 0.88 (5)	7.42 ± 3.79 (5)	0.38 ± 0.20 (5)	4.95 ± 2.30 (5)	0.21 ± 0.11 (5)

*P < 0.05 versus CRLP+

**P < 0.005 versus CRLP+.

## Abbreviations

apoE: Apolipoprotein E;
CRLP: Chylomicron remnant-like particles;
FCH: Free cholesterol;
HMDM: Human monocyte-derived macrophages;
IgG: Immunoglobulin G;
IMDM: Iscove's Dulbecco's modified medium;
LPL: Lipoprotein lipase;
PL: Phospholipid;
sPLA_2_: Secretory phospholipase A2;
TCH: Total cholesterol;
TG: Triacylglycerol.
